# Editorial: Myelin Repair: At the Crossing-Lines of Myelin Biology and Gene Therapy

**DOI:** 10.3389/fncel.2022.853742

**Published:** 2022-02-11

**Authors:** Dominik Fröhlich, Dominic J. Gessler, Matthias Klugmann

**Affiliations:** ^1^Translational Neuroscience Facility, School of Medical Sciences, UNSW Sydney, Kensington, NSW, Australia; ^2^Horae Gene Therapy Center, University of Massachusetts Chan Medical School, Worcester, MA, United States; ^3^Department of Microbiology and Physiological Systems, University of Massachusetts Chan Medical School, Worcester, MA, United States; ^4^Department of Neurosurgery, University of Minnesota, Minneapolis, MN, United States

**Keywords:** myelin, gene therapy, leukodystrophy, white matter, oligodendrocytes

Leukodystrophies are genetic diseases characterized by impaired formation or maintenance of the brain's white matter (myelin). Although considered rare, over 50 different Leukodystrophies are known, amounting to a total population incidence of one in 7,600 births (Bonkowsky et al., 2010).

Premature demise is commonly the consequence of neurodevelopmental deficits caused by poor myelin formation. The high mortality, lack of treatment options, and monogenic nature of many white matter disorders make them particularly amenable to gene therapy.

In fact, gene therapy employing an *ex vivo* approach has shown remarkable clinical success for X-linked adrenoleukodystrophy (X-ALD) and metachromatic leukodystrophy (MLD) (Biffi et al., 2011). Intuitively, mutations of genes encoding proteins essential for oligodendrocyte functions underly many white matter disorders. However, *in vivo* gene therapy directed at oligodendrocytes has been notoriously difficult amid the lack of efficient gene delivery systems targeting glia.

Myelin dysfunction can also be secondary to cellular or metabolic pathologies of astroglia or neurons reflecting abnormal glia-neuronal interactions. To improve our understanding of the complex pathophysiologies and disease etiologies, leukodystrophy models are needed that foster the development of therapies and the design of clinical trials.

This Research Topic compiles original research and review articles from leading scientists in myelin biology and gene therapy, ranging from generating novel genetic cell and animal models to developing experimental gene therapy strategies for white matter disorders, for which some are currently in clinical trial.

In an original research article, Shaker et al. describe a fast and efficient new protocol to grow human brain organoids comprising myelinating oligodendrocytes, cortical neurons, and astrocytes in 42 days. These myelinated brain organoids are a valuable tool to study CNS disorders associated with myelin defects and will be instrumental for drug discovery and developing novel therapeutics.

The work by Fröhlich et al. characterizes humanized rodent models for the autosomal recessive disorder Hypomyelination with Brain stem and Spinal cord involvement and Leg spasticity (HBSL), which was first described in 2013 (Taft et al., 2013). Using CRISPR/Cas9, the authors introduced the HBSL-causing *Dars1*^*D*367*Y*^ point mutation into the mouse genome. Homozygous *Dars1*^*D*367*Y*^ mice were phenotypically normal, which was overcome by generating compound heterozygous *Dars1*^*D*367*Y*/*null*^ mice. These mice showed neurological signs similar to HBSL patients with the corresponding missense mutation, enabling future therapeutic proof-of-concept studies.

In addition, this Research Topic provides updates on the latest pathophysiology and therapy development for traumatic CNS injury, megalencephalic leukoencephalopathy, POLR3-related leukodystrophy, HBSL, LBSL, GM1 and GM2 gangliosidosis, and Canavan disease.

Traumatic brain and spinal cord injuries often result in demyelination and the failure to remyelinate. Huntemer-Silveira et al. review the effects of traumatic CNS injury on oligodendrocytes and the consequences of disrupted endogenous remyelination mechanisms. The authors highlight recent rodent and clinical studies aimed at enhancing remyelination through therapies involving small molecules, RNA interference, monoclonal antibodies, and cell replacement strategies.

Megalencephalic leukoencephalopathy with subcortical cysts (MLC) is a vacuolating leukodystrophy characterized by megalencephaly, loss of motor function, epilepsy, mild mental decline, and no available treatment options. Bosch and Estevez provide a detailed overview of pathophysiology, established animal models, and potential therapeutic strategies, emphasizing preclinical adeno-associated virus (AAV)-MLC1 gene therapy in Mlc1 knockout mice from the same lab.

POLR3-related leukodystrophy is one of the most common types of hypomyelinating leukodystrophy, also known as 4H leukodystrophy, manifesting with hypomyelination, hypodontia, and hypogonadotropic hypogonadism. Perrier et al. explore in their review article the use of stem cell transplantation, gene replacement therapy, and gene editing as avenues for future treatment options.

The leukodystrophies Leukoencephalopathy with Brainstem and Spinal cord involvement and Lactate elevation (LBSL) and HBSL are both spectrum disorders characterized by a similar clinical presentation and the lack of curative treatment options. LBSL and HBSL are caused by mitochondrial and cytoplasmic aspartyl-tRNA synthetase mutations, respectively. Muthiah et al. highlight similarities and differences between the two leukodystrophies and summarize current knowledge of preclinical and clinical features, including neuroimaging, diagnosis, disease mechanisms, mouse models, and treatment options.

GM1 and GM2 gangliosidosis are devastating neurodegenerative lysosomal storage disorders with white matter manifestation. The development of gene therapies for GM1 and GM2 gangliosidosis has seen promising progress in recent preclinical studies. These and other findings are outlined in the review article by Maguire and Martin, which critically discusses current viewpoints on the origin of white matter deficits in gangliosidoses and potential obstacles for effective gene therapies.

An essential prerequisite for successfully treating leukodystrophies is to enable myelin repair in a time-dependent fashion to restore CNS homeostasis. Using Canavan disease as a model system, Lotun et al. review the role of N-acetylaspartate (NAA), one of the most abundant metabolites in the mammalian CNS, in the normal and diseased brain and discuss the involvement of astrocytes in both Canavan disease and other leukodystrophies.

To conclude this Research Topic, von Jonquieres et al. provide a comprehensive overview of emerging gene therapy concepts, specifically targeting glial cells. The authors review the latest advances in genetic and cellular treatment strategies for leukodystrophies, including *ex vivo* stem cell gene therapy and the use of AAV *in vivo*.

## Author Contributions

All authors listed have made a substantial, direct, and intellectual contribution to the work and approved it for publication.

## Conflict of Interest

MK is affiliated with Research Beyond Borders, Boehringer-Ingelheim GmbH and Co KG. DG is affiliated with ASPA Therapeutics, Inc. The remaining author declares that the research was conducted in the absence of any commercial or financial relationships that could be construed as a potential conflict of interest.

## Publisher's Note

All claims expressed in this article are solely those of the authors and do not necessarily represent those of their affiliated organizations, or those of the publisher, the editors and the reviewers. Any product that may be evaluated in this article, or claim that may be made by its manufacturer, is not guaranteed or endorsed by the publisher.
